# The association of depression following percutanous coronary intervention with adverse cardiovascular events: Protocol for a systematic review and meta-analysis

**DOI:** 10.1097/MD.0000000000013952

**Published:** 2019-01-11

**Authors:** Yanfei Liu, Yinke Zhao, Jinfan Tian, Tiejun Tong, Rui Gao, Yue Liu

**Affiliations:** a Graduate School of Beijing University of Chinese Medicine; b Cardiovascular diseases center, Xiyuan hospital of China academy of Chinese medical sciences; c Institute of Clinical Pharmacology of Xiyuan Hospital, China Academy of Chinese Medical Sciences, Beijing; d School of Chinese Medicine, Li Ka Shing Faculty of Medicine, The University of Hong Kong, Pokfulam, Hong Kong, China; e Department of Cardiology, Beijing Anzhen Hospital, Capital Medical University, Beijing, China; f Department of Mathematics, Hong Kong Baptist University, Kowloon Tong, Hong Kong, China.

**Keywords:** adverse cardiovascular events, depression, percutanous coronary intervention, systematic review

## Abstract

**Introduction:**

Percutanous coronary intervention (PCI) has been increasingly used for patients suffered from severe coronary artery disease. However, physical trauma and potential adverse events related to the procedure often result in detrimental psychological stress. Accumulating evidences have shown that depression is closely related to coronary artery disease. However, the association of depression following percutanous coronary intervention with adverse cardiovascular events is still unknown.

**Objective:**

This review is designed to assess the prognostic association of depression following PCI with adverse cardiac events.

**Methods and analysis:**

The following databases will be searched, PubMed, the EMBASE, CINAHL and Web of Science of English-language publications from inception to 30 October 2018. Cross-referencing from retrieved studies will be conducted additionally, and observational studies were included. Two independent review authors will do the study selection on the basis of the study eligibility criteria. Extracted data will be used for quantitative and qualitative evidence synthesis as well as to assess methodological quality of studies using the Newcastle-Ottawa checklist. The primary objective of this review is adverse cardiac events, presented as a composition of myocardial infarction, repeat coronary revascularization, cardiac readmission, and cardiac death. The accumulated evidence is evaluated and graded according to Grading of Recommendations, Assessment, Development and Evaluation (GRADE).

**Results and conclusions:**

This review will explain the association of depression following percutanous coronary intervention with adverse cardiovascular events, and provide physicians with scientific evidence for psychological intervention in patients after PCI.

**Prospero registration number:**

CRD42018112486.

## Abstract 2

Supplemental Digital Content is available in the text

## 1 Introduction

It is well known age, gender, diabetes, and hypertension are greatly contributing to cardiac events such as myocardial infarction, repeat revisualization procedure, cardiac death, and cardiac readmission.^[1–3]^ The morbidity and mortality of Cardiac events are increasing worldwide.^[4]^ Percutaneous coronary intervention (PCI), which is a safe and effective treatment, has been increasingly used for patients with significant coronary artery disease.^[5,6]^ However, physical trauma and potential adverse events related to the procedure often result in significant psychological stress.

It has been shown that depression as negative emotion is related to development of coronary heart disease (CHD).^[7,8]^ Whereas coronary artery intervention aggravate patients’ depression.^[9]^ There is a high prevalence of depression among patients who suffered from an acute cardiac event or underwent PCI.^[10]^ While observational studies assessed relationship of depression with adverse cardiovascular events following percutanous coronary intervention have reached diverse conclusions. Several studies concluded that depression is predictive of mortality or adverse cardiac events in patient post-PCI.^[11,12]^ While other studies showed that there were no association between depression and mortality after myocardial infarction.^[13–15]^ The previous meta analysis has reported that post-MI depression is associated with an increased risk of adverse cardiovascular outcomes.^[16,17]^ However, the association between depression following PCI and adverse cardiovascular events was not mentioned in the 2 earlier meta-analyses. The association of depression following PCI with adverse cardiovascular events is still unknown. Thus, it is of importance to reassess association of depression following PCI with adverse cardiovascular events. The present analysis focuses on the prognostic association of depression following PCI with adverse cardiac events.

## 2 Outcomes

The primary outcome of this review is adverse cardiac events, presented as a composition of myocardial infarction, repeat coronary revascularization, cardiac readmission and cardiac death. Mortality was the secondary endpoint.

## 3 Methods

### 3.1 Standards

The protocol was developed in accordance with the Preferred Reporting Items for Systematic Reviews and Meta-Analysis (PRISMA) statements and the guidelines in Cochrane Handbook for systematic Reviews of interventions.^[18]^ A PRISMA-P checklist is attached (supplementary file1)

### 3.2 Protocol and registration

This systematic review protocol has been registered with the PROSPERO (CRD42018112486).

### 3.3 Eligibility criteria

The studies were considered to be eligible if they met the following inclusion criteria:

1. Observational study;
2. adults ≥ 18 years of age;
3. contained patients diagnosed with depression within 6 months after PCI (including during hospitalization), using reliable and validated instruments^[19–21]^ to assess depression;
4. studies included adverse cardiac events (including recurrent myocardial infarction (MI), repeat coronary revascularization, cardiac readmission and cardiac death) and/or mortality as outcomes.
5. All patients were followed up for more than 1 year after PCI.
6. Published in English up to 30 October 2018.

### 3.4 Information sources

The following databases will be searched, PubMed, the EMBASE, CINAHL and Web of Science of English-language publications from inception to 30 October 2018. Cross-referencing from retrieved studies will be conducted additionally.

### 3.5 Search strategy

A preliminary search strategy for PubMed is demonstrated in Table 1. Search term will be adapted to other databases based on the specific requirements for each database. We also manually checked reference lists to identify other potential studies.

**Table 1:**
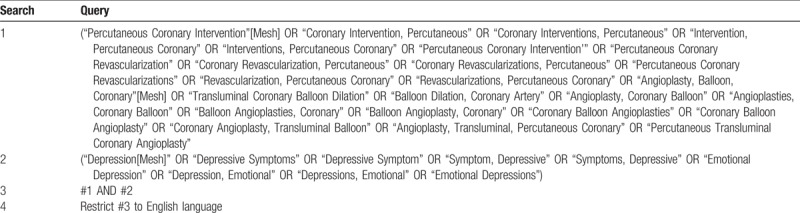
Preliminary search strategy: PubMed format.

### 3.6 Data management

All the literature search results will be combined and uploaded to one single EndNote (X.8) library. Duplicates will be removed.

### 3.7 Selection process

**Figure 1:**
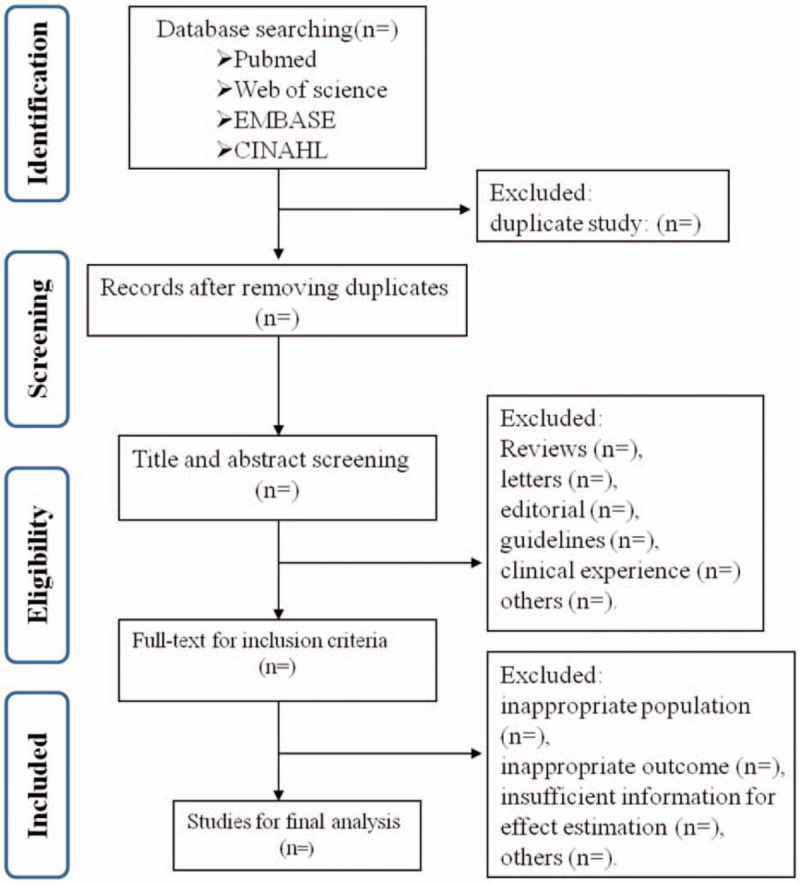
Flow chart of literature screening process.

Two independent review authors will do the study selection on the basis of the study eligibility criteria. The study selection will be accomplished via 2 stages. Firstly, all the titles and abstracts will be screened by 2 researchers independently, to obtained which appear to meet the inclusion criteria or there is any uncertainty. Any discrepancies will be resolved by discussion. Reviews, letters or editorial, guidelines or clinical experience were excluded. Next, both authors will then obtain the full-text articles to further identified these meet the inclusion criteria. Reasons for exclusion of articles in the full-text screening session will be documented as follows, inappropriate population, inappropriate outcome, insufficient information for effect estimation, others. Discrepancies will be discussed by 2 authors. Further consultation with a 3rd review will be carried out if consensus cannot be reached. A proposed flow chart shown in Figure 1, illustrates the whole search process.

### 3.8 Data extraction

All the required data will be double extracted by 2 authors using a standardized data extraction form. Data in detail was extracted from each individual study that ultimately was included in the present study, including the following:

1. the 1st author's name and publication year;
2. study design;
3. patient selection;
4. participants’ age;
5. percentage of females;
6. instrument prevalence;
7. assessment timing;
8. clinical outcomes;
9. mean follow-up time
10. risk factor adjustment.

Attempt was made to contact authors who published their studies only with abstract, but, failing to obtain original data, these studies were excluded from the present study.

### 3.9 Quality appraisal for included studies

The methodological quality of studies was assess using the Newcastle–Ottawa checklist,^[22]^ with the highest score (the score meaning better quality) being 9. We defined the high quality as ≥7 scores, medium quality as 4 to 6 scores, and low quality as < 4 scores. Newcastle-Ottawa checklist is attached (supplementary file 2).

The 2 authors extracted data independently. Upon completion, they will then review the results of assessment. Any disagreement was resolved by consulting with the 3rd investigator. Summary risk of bias table will be produced.

### 3.10 Statistical analysis

If there is sufficient evidence, we will consider conducting a meta-analysis to estimate the pooled effect. All statistical analysis was performed using STATA software. Odds ratio (OR) and 95% confidence intervals (CI) were used as summary statistics. Heterogeneity across studies was analyzed using *I*^*2*^ [*I*^*2*^=Q-df]/Q; where Q is the Chi-square statistic and df is the degree of freedom]. There were no statistical heterogeneity if *P≥.1* and *I*^*2*^* ≤50%,* and fix-effect model was used for meta-analysis. *I*^*2*^ *>* *50* indicates the possibility of heterogeneity among the studies, and random-effects model was used. Sources of heterogeneity were explored by sensitivity analysis. If *I*^*2*^ was larger than 75%, which means there was obviously statistical heterogeneity among studies, only results from each single study were present respectively rather than pooling analysis. Begger's plot analysis was used to detect publication bias. If these are not possible we will discuss possible sources of bias across studies.

If we find selected studies bearing sufficiently homogenous, our narrative synthesis will also described subgroup analyses, including: type of depression instrument; population characteristics, such as age strata and gender; number of subjects per study (dichotomized smaller number vs. larger number).

### 3.11 Confidence in cumulative evidence

The strength of evidence will be assessed by the GRADE (Grades of Recommendation, Assessment, Development and Evaluation) approach for the relating domains, such as risk of bias, the methodological flaws, the consistency of results, precision and publication bias.^[23]^ Studies will be rated as high (future evidence is unlikely to change the conclusion obtained from our research), moderate (further studies might alter our conclusion), low (further evidence is needed to answer the involved research question with increased confidence), very low quality (the estimate of effects is very uncertain).

### 3.12 Ethics and dissemination

Ethical review is not required as this protocol is for a systematic review. The result will be published in a peer-review journal. This review will the explain relationship between depression following PCI and cardiovascular events, and provide physicians with scientific evidence for psychological intervention in patients after PCI.

## 4 Discussion

The previous meta analysis has reported that post-MI depression is associated with an increased risk of adverse cardiovascular outcomes.^[16,17]^ However, the association of depression following PCI with cardiovascular events is still unknown. Currently, although not all the mechanisms have been recognized, several reasons for the prognostic value of depression in patients undergoing PCI were established. First, depression is closely related to unhealthy lifestyles^[24]^ and compromised adherence to medication for coronary heart disease,^[25]^ which, consequently, contributes to the adverse cardiac events following PCI. Second, from pathophysiological perspective, depression is associated with neuroendocrine dysfunction, inflammation, impaired endothelial function, and nutritional deficiencies.^[26]^ The compromised left ventricular function resulted from disorder of the autonomic nervous system and serious myocardial remodeling after depressed lead to the poor prognosis after PCI.^[27]^ Reid G J et al^[28]^ reported that experimentally induced mental stress induced platelet activation in patients with coronary artery disease. Third, depression and adverse cardiac events post-PCI share common risk factors, such as current smoking,^[29]^ central obesity, hypercholesterolemia and diabetes.^[30,31]^ It has been shown that depressed patients also suffer from glucose and lipid metabolism disorder, common risks factor for adverse cardiac events post PCI.

According to Milani, R V et al^[32]^ depressed patients who completed rehabilitation had a 73% lower mortality (8% vs 30%; *P* = .0005) compared with control depressed subjects who did not complete rehabilitation. Evidences have shown that cardiac rehabilitation^[33]^ and pre-discharged counseling have the ability to improve the prognosis of cardiovascular disease. Janey C. Petersona et al first reported that a threshold in physical activity in CAD patients with depressive is associated with a reduction in cardiovascular morbidity and mortality via enhancing parasympathetic tone and decreasing inflammation.^[34]^ Accumulating evidences have shown that depression is closely related to coronary artery disease. However, the association between adverse cardiovascular events after PCI and depression is still unknown. Thus, it is of importance to reassess relationship of depression with adverse cardiovascular events following PCI. The present analysis focuses on the prognostic association of depression with adverse cardiac events following PCI.

## Author contributions

**Conceptualization:** Rui Gao, Yue Liu.

**Funding acquisition:** Yue Liu.

**Investigation:** Yanfei Liu, Jinfan Tian.

**Methodology:** Yanfei Liu, Yinke Zhao, Tiejun Tong, Yue Liu.

**Project administration:** Rui Gao.

**Software:** Tiejun Tong.

**Supervision:** Rui Gao, Yue Liu.

**Writing – original draft:** Yanfei Liu, Jinfan Tian, Yue Liu.

**Writing – review & editing:** Yanfei Liu, Yinke Zhao, Yue Liu.

Yue Liu orcid: 0000-0002-0084-863X.

## Supplementary Material

Supplemental Digital Content

## References

[1] Stähli Barbara E Wischnewsky Manfred B Jakob Philipp Gender and age differences in outcomes of patients with acute coronary syndromes referred for coronary angiography. Catheter Cardiovasc Interv 2018;doi: 10.1002/ccd.27712. [Epub ahead of print]. 10.1002/ccd.27712 30291678

[2] Bazdyrev ED Polikutina OM Kalichenko NA Pulmonary function in patients with type 2 diabetes and coronary artery disease. Klin Med (Mosk) 2016;94:366–73. 30289649

[3] Cole Nicholas I Suckling Rebecca J Swift Pauline A The association between serum sodium concentration, hypertension and primary cardiovascular events: a retrospective cohort study. J Hum Hypertens 2018;2018: doi: 10.1038/s41371-018-0115-5. [Epub ahead of print]. 10.1038/s41371-018-0115-5 30250270

[4] Kochanek K Xu J Murphy S Deaths:final data for 2009. Natl Vital Stat Rep 2011;60:111–7. 24974587

[5] Park HW Yoon CH Kang SH Early- and late-term clinical outcome and their predictors in patients with ST segment elevation myocardial infarction and non-ST-segment elevation myocardial infarction. Int J Cardiol 2013;169:254–61. 24071385 10.1016/j.ijcard.2013.08.132

[6] Jaffery Z Prasad A Lee JH Drug-eluting coronary stentsVfocus on improved patient outcomes. Patient Relat Outcome Meas 2011;2:161–74. 22915977 10.2147/PROM.S24796 PMC3417932

[7] Vogelzangs N Seldenrijk A Beekman AT Cardiovascular disease in persons with depressive and anxiety disorders. J Affect Disord 2010;125:241–8. 20223521 10.1016/j.jad.2010.02.112 PMC2964458

[8] O’Neil A Fisher Aaron J Kibbey Katherine J Depression is a risk factor for incident coronary heart disease in women: an 18-year longitudinal study. J Affect Disord 2016;196:117–24. 26921864 10.1016/j.jad.2016.02.029

[9] Zhang P Study of anxiety/depression in patients with coronary heart disease after percutaneous coronary intervention. Cell Biochem Biophys 2015;72:503–7. 25575895 10.1007/s12013-014-0495-2

[10] Gu G Zhou Y Zhang Y Increased prevalence of anxiety and depression symptoms in patients with coronary artery disease before and after percutaneous coronary intervention treatment. BMC Psychiatry 2016;16:259. 27450548 10.1186/s12888-016-0972-9 PMC4957885

[11] Damen Nikki L Versteeg Henneke Boersma Eric Depression is independently associated with 7-year mortality in patients treated with percutaneous coronary intervention: results from the RESEARCH registry. Int J Cardiol 2013;167:2496–501. 22560933 10.1016/j.ijcard.2012.04.028

[12] van Dijk Milan R Utens Elisabeth MWJ Dulfer Karolijn Depression and anxiety symptoms as predictors of mortality in PCI patients at 10 years of follow-up. Eur J Prev Cardiol 2016;23:552–8. 25665581 10.1177/2047487315571889

[13] Lane D Carroll D Ring C Effects of depression and anxiety on mortality and quality-of-life 4 months after myocardial infarction. J Psychosom Res 2000;49:229–38. 11119779 10.1016/s0022-3999(00)00170-7

[14] Lane D Carroll D Ring C In-hospital symptoms of depression do not predict mortality 3 years after myocardial infarction. Int J Epidemiol 2002;31:1179–82. 12540719 10.1093/ije/31.6.1179

[15] Kurdyak Paul A Gnam William H Goering Paula The relationship between depressive symptoms, health service consumption, and prognosis after acute myocardial infarction: a prospective cohort study. BMC Health Serv Res 2008;8:200. 18826611 10.1186/1472-6963-8-200 PMC2576230

[16] van Melle Joost P de Jonge Peter Spijkerman Titia A Prognostic association of depression following myocardial infarction with mortality and cardiovascular events: a meta-analysis. Psychosom Med 2004;66:814–22. 15564344 10.1097/01.psy.0000146294.82810.9c

[17] Meijer Anna Conradi Henk Jan Bos Elisabeth H Prognostic association of depression following myocardial infarction with mortality and cardiovascular events: a meta-analysis of 25 years of research. Gen Hosp Psychiatry 2011;33:203–16. 21601716 10.1016/j.genhosppsych.2011.02.007

[18] Shamseer L Moher D Clarke M Preferred reporting items for systematic review and meta-analysis protocols (PRISMA-P) 2015: elaboration and explanation. BMJ 2015;350:7647. 10.1136/bmj.g7647 25555855

[19] Zigmond AS Snaith RP The hospital anxiety and depression scale. Acta Psychiatr Scand 1983;67:361–70. 6880820 10.1111/j.1600-0447.1983.tb09716.x

[20] Beck AT Steer RA Brown GK Manual for the revised Beck depression inventory. Texas Psychological Corporation, San Antonio 1987.

[21] Kroenke K Spitzer RL Williams JB The PHQ-9: validity of a brief depression severity measure. J Gen Intern Med 2001;16:606–13. 11556941 10.1046/j.1525-1497.2001.016009606.x PMC1495268

[22] Wells GA Shea B O’Connell D: The Newcastle-Ottawa Scale (NOS) for assessing the quality of nonrandomised studies in MetaAnalyses, 2014 Available at: http://www.ohri.ca/programs/clinical_epidemiology/oxford.asp Accessed October 1, 2017.

[23] Balshem H Helfand M Schunemann HJ GRADE guidelines: 3. Rating the quality of evidence. J Clin Epidemiol 2011;64:401–6. 21208779 10.1016/j.jclinepi.2010.07.015

[24] Benyamini Yael Roziner Ilan Goldbourt Uri Depression and anxiety following myocardial infarction and their inverse associations with future health behaviors and quality of life. Ann Behav Med 2013;46:310–21. 23645421 10.1007/s12160-013-9509-3

[25] Feng Can Ji Tao Liu Yu Role of depression in secondary prevention of Chinese coronary heart disease patients receiving percutaneous coronary intervention. PLoS One 2017;12:e0187016. 29267269 10.1371/journal.pone.0187016 PMC5739347

[26] Munk Peter Scott Isaksen Kjetil Brønnick Kolbjørn Symptoms of anxiety and depression after percutaneous coronary intervention are associated with decreased heart rate variability, impaired endothelial function and increased inflammation. Int J Cardiol 2012;158:173–6. 22575625 10.1016/j.ijcard.2012.04.085

[27] Shi Shaobo Liang Jinjun Liu Tao Depression increases sympathetic activity and exacerbates myocardial remodeling after myocardial infarction: evidence from an animal experiment. PLoS One 2014;9:e101734. 25036781 10.1371/journal.pone.0101734 PMC4103791

[28] Reid Graham J Seidelin Peter H Kop Willem J Mental-stress-induced platelet activation among patients with coronary artery disease. Psychosom Med 2009;71:438–45. 19251865 10.1097/PSY.0b013e31819cc751

[29] Lehto S Koukkunen H Hintikka J Depression after coronary heart disease events. Scand Cardiovasc J 2000;34:580–3. 11214011 10.1080/140174300750064512

[30] Vural M Acer M Akbas B The scores of Hamilton depression, anxiety, and panic agoraphobia rating scales in patients with acute coronary syndrome. Anadolu Kardiyol Derg 2008;8:43–7. 18258533

[31] Pogosova Nana Kotseva Kornelia De Bacquer Dirk Psychosocial risk factors in relation to other cardiovascular risk factors in coronary heart disease: Results from the EUROASPIRE IV survey. A registry from the European Society of Cardiology. Eur J Prev Cardiol 2017;24:1371–80. 28534422 10.1177/2047487317711334

[32] Milani RV Lavie CJ Impact of cardiac rehabilitation on depression and its associated mortality. Am J Med 2007;120:799–806. 17765050 10.1016/j.amjmed.2007.03.026

[33] Milani RV Lavie CJ Cassidy MM Effects of cardiac rehabilitation and exercise training programs on depression in patients after major coronary events. Am Heart J 1996;132:726–32. 8831359 10.1016/s0002-8703(96)90304-x

[34] Peterson Janey C Charlson Mary E Wells Martin T Depression, coronary artery disease, and physical activity: how much exercise is enough? Clin Ther 2014;36:1518–30. 25456561 10.1016/j.clinthera.2014.10.003 PMC4311731

